# Advances in the Starling Principle and Microvascular Fluid Exchange; Consequences and Implications for Fluid Therapy

**DOI:** 10.3389/fvets.2021.623671

**Published:** 2021-04-06

**Authors:** Thomas E. Woodcock, C. Charles Michel

**Affiliations:** ^1^Fluid Physiology, Stockbridge, Hampshire, United Kingdom; ^2^Department of Bioengineering, Imperial College London, London, United Kingdom

**Keywords:** resuscitation, crystalloid and colloid infusion, hypoalbuminaemia, fluid therapy, oedema (edema), starling principle, glycocalyx model

## Abstract

Ernest Starling first presented a hypothesis about the absorption of tissue fluid to the plasma within tissue capillaries in 1896. In this Chapter we trace the evolution of Starling's hypothesis to a principle and an equation, and then look in more detail at the extension of the Starling principle in recent years. In 2012 Thomas Woodcock and his son proposed that experience and experimental observations surrounding clinical practices involving the administration of intravenous fluids were better explained by the revised Starling principle. In particular, the revised or extended Starling principle can explain why crystalloid resuscitation from the abrupt physiologic insult of hypovolaemia is much more effective than the pre-revision Starling principle had led clinicians to expect. The authors of this chapter have since combined their science and clinical expertise to offer clinicians a better basis for their practice of rational fluid therapy.

## Introduction

Hippocrates and Aristotle made reference to lymphatic vessels more than 2,000 years ago, and the Greek physician Galen had observed mesenteric chyle in his dissections of living animals in the second century AD. Gasparo Aselli was the Italian anatomist (1581–1626) whose studies on living dogs in 1622 rediscovered the appearance of mesenteric lacteals (milky veins) after feeding. Aselli's glands are para-aortic lymph nodes near the pancreas of mammals. Danish physician Thomas Bartholin and Olaus Rudbeck, who was Professor of Anatomy, Biology and Physics at Uppsala University in Sweden published the first full description of human lymphatics in 1652. Bartholin named the vessels he observed vasae lymphaticae, while in Sweden Olauf Rudbeck had made unpublished presentations on his own discovery. In mid-century Britain, William Hewson observed the rhythmic contraction of lymphatic vessels and William Hunter proposed that the purpose of the lymphatic system is to absorb tissue fluids for return to the blood circulation (1–3). The work of Ernest Starling in late-nineteenth century London gave us an appreciation of the Starling forces that result in the formation of extracellular fluid and lymph by capillary filtration from the plasma of circulating blood. Starling not only confirmed that raising microvascular pressures increased interstitial fluid formation, as indicated by Ludwig & Noll, (4) but also demonstrated in perfused hind limbs of anesthetized dogs that saline solution injected into the tissues could be absorbed directly into the blood, whereas injections of plasma into the tissues could not be absorbed (5). He suggested the balance of hydrostatic and colloid osmotic pressures across microvascular (i.e., capillary and venular) walls was responsible not only for the partitioning of fluid between plasma and tissues but also for rapid adjustments of blood volume. Starling reviewed his work and its implications in Schafer's Textbook and suggested the general role of the lymphatics was to clear excess fluid remaining in the tissues. In his conclusion, he noted that since the hydrostatic pressure at the arterial end of capillaries was greater than that at the venous end, fluid might be filtered from plasma to tissues at the arterial end and reabsorbed at the venous end (1). This picture was soon adopted and continued to be used for the next 100 years as a popular way of teaching Starling's Hypothesis. There is no reproducible experimental evidence for this picture and the recognition that microvascular walls are permeable to plasma proteins means a picture of this kind could only exist transiently. Until recently standard textbooks presented the lymphatics as an accessory route through which fluid can flow from the interstitial spaces into the blood (6). Levick's Introduction to Cardiovascular Physiology 6e dismisses this concept as “still taught tenaciously… yet clearly disproved by a large body of evidence over the past 20 years.” (7) Tissue fluid balance in most tissues critically depends on lymphatic function. In this chapter we introduce the twenty-first century view of Starling forces and the vital circulations of extracellular fluids pumped by the lymphatic system.

### Starling Forces

We start from Starling's classic experiments on dogs, published in 1896 (4). In one experiment he created ‘artificial oedema’ with 1% Sodium Chloride solution injected into the hind leg of a freshly-killed dog. The result enabled him to “affirm with certainty that isotonic salt solutions can be taken up directly by the blood circulating in the blood vessels.” This fundamental physiological principle is unquestioned to this day and relied upon when fluids are administered subcutaneously in small animal veterinary practice.

More than a half century later, Staverman (8) suggested that osmotic pressures exerted by solutions across membranes which were permeable to solutes, could be better understood in terms of Onsager's theory of irreversible thermodynamics. Onsager's theory predicted that flows of solvent (water) and solute through the membrane should interact, modifying the forces maintaining the process (i.e. the forces being the differences in hydrostatic and osmotic pressures across the membrane). Thus, the effective osmotic pressure difference exerted by solutions at different concentrations across a leaky membrane would be less than that exerted by the same solutions across a perfect semi-permeable membrane by a factor, σ. A perfect semi-permeable membrane is one that is permeable to the solvent (water) yet completely impermeable to the solute. The fraction of solute carried by the solvent through a leaky membrane during ultrafiltration was the complement of σ that is (1-σ). The value of σ varies between 0 and 1.0, where σ =1 describes a perfectly semi-permeable membrane and σ = 0 indicates a membrane which hinders the passage of solute through it no more than the passage of water. This concept was developed and applied to biological membranes by Kedem and Katchalsky (9) who discussed the osmotic pressures exerted by small molecules across microvascular walls in an attempt to measure concentration differences for small solutes. Kedem & Katchalsky's equation for fluid flow across biological membranes is what we know as the Starling equation. For fluid exchange across microvascular walls it is written as:

(1) $$\begin{array}{l} J_{V}\;=K\left(\Delta P\;-\;\sigma \Delta \Pi \right). \end{array}$$

Here, J_*V*_ is the fluid filtration rate (ml.min^−1^); Δ*P* is the hydrostatic pressure difference across microvascular walls, ΔΠ the colloid osmotic pressure difference and σ is Staverman's reflection coefficient. Strictly speaking the colloid osmotic pressure of plasma, σΔΠ is the summation of the products of all the plasma solutes contributing to the total osmotic pressure difference across microvascular walls, that is, $\underset{n}{\sum }\sigma \Delta \Pi$. Because the reflection coefficients of the small solutes are low (<0.1) and their concentration differences across microvascular walls negligible, the colloid osmotic pressure is the osmotic pressure exerted across microvascular walls by plasma macromolecules.

The constant, K, as used here, is the hydraulic conductance of microvascular walls to fluid and is dependent on both their permeability coefficient and their surface area for filtration. In many tissues, the area for fluid exchange can be rapidly varied by vasomotor reflexes. Woodcock proposes that clinicians may find it easier to think of conductance (or conductivity or permeability) of a capillary wall to the passage of solvent as a transendothelial resistance (10). To be precise, trans-endothelial resistance is the inverse of the product of hydraulic conductivity Lp and endothelial surface area A through which flow is occurring, that is, (1/LpA) rather than (1/Lp).

The Starling equation remains valid today (11). Tissue fluid balance, plasma volume regulation and clinical oedema formation are governed by the Starling principle of microvascular fluid exchange. The classical Starling hypothesis assumed that σ = 1, since Starling believed microvascular walls were completely impermeable to plasma proteins. Consider as an example the blood-brain barrier which is almost impermeable to both small molecules and large molecules, expressed in the reflection coefficient σ for mannitol and the reflection coefficient σ for albumin being close to 1. Clinicians could therefore use intra-vascular injection of a solution as an osmotic diuretic to reduce brain oedema. By contrast, the microvasculature of the sinusoidal tissues poses little or no restriction on the movement of smaller or larger molecules, and reflection coefficients here approach zero. Fluid exchange depends on hydrostatic pressure differences.

### Mean Capillary Pressure

The axial capillary pressure gradient and the mean capillary pressure are determined by the local arterial and venous pressures and the ratio of the capillary inflow (arteriolar) resistance to the capillary outflow (venular) resistance (12). This means that if the arterial pressure and blood flow are raised (e.g., by increased cardiac output) then providing the local inflow and outflow resistances are unchanged, the mean capillary pressure is raised also. Similarly, if resistances are unchanged and the arterial pressure is lowered, capillary pressure will fall. In the systemic circulation, however, tissue blood flow is continually adjusted by cardiovascular reflexes and local mechanical and chemical responses to ensure tissue oxygenation is adequate. Cardiovascular reflexes maintaining mean arterial pressure may increase arteriolar resistance to resting skeletal muscle reducing the tissue blood flow and lowering mean capillary pressure while arterial pressure is increased. Detailed investigations into the response and their effects on net fluid exchange were explored by Folkow and his colleagues in the 1960s (13, 14). An interesting comparison between the responses of cats and humans is reported by Mellander et al. (15).

Intravascular hypervolaemia causes capillary hypertension, while hypovolaemia causes capillary hypotension. Indeed, the compensatory arteriolar constriction that accompanies hypovolaemia amplifies the associated capillary hypotension. It is important to realize that the vasodilation of systemic inflammatory response has the effect of simultaneously lowering the arterial blood pressure and raising the mean capillary pressure.

***Extravascular colloid osmotic pressure*** is the second filtration-driving Starling force. Techniques were developed during the twentieth century to sample interstitial fluid and to measure interstitial colloid osmotic pressure *in-vivo*. π_i_ was found to be greater than earlier workers had believed. Levick (16) reviewed data from a range of different tissues in mammals, including humans, in 1991 and showed that the Starling equation predicted levels of filtration into the tissues that were many times greater than could be consistent with lymph flow from the tissues, even if the microvascular pressure were equal to the venous pressure.

It is now recognized that the luminal endothelial glycocalyx is the molecular filter of both continuous and fenestrated endothelia and may contribute as much as half of the hydraulic resistance to flow to the intact microvascular wall (17, 18). Filtered fluid leaving the glycocalyx creates a subdomain of the interstitial fluid with a lower colloid osmotic pressure than that of the average interstitial fluid. This subdomain is the sub-glycocalyx space. Solvent filtered by the glycocalyx of continuous endothelia is channeled into the intercellular clefts and through occasional breaks in the tight junction. The velocity of solvent flow through these inter-endothelial channels is very high and the volume contained within this subdomain is very small. Calculations by Michel (19) and Weinbaum (20) revealed that even when the total pressure difference across the microvascular wall was as little as 1 cm H_2−_O, the filtration velocity through the channels would prevent back diffusion of macromolecules into the sub-glycocalyx space from the main interstitial space. Consequently, the colloid osmotic pressure of fluid immediately beyond the endothelial glycocalyx layer (in the subglycocalyx space) is less than the general interstitial fluid because macromolecules are largely excluded by the glycocalyx, and varies inversely with the transendothelial filtration rate. Most plasma macromolecules cross microvascular walls through openings in the glycocalyx leading to trans-endothelial channels or transcytotic vesicles that discharge their contents to the interstitium (21–25). The colloid osmotic pressure of fluid in the subglycocalyx space is reduced at high filtration rates and increases when filtration rate falls. While it has been recognized for over 60 years that the main pathway for macromolecules was separate from that for water and small hydrophilic solutes, it was 30 years before it's implication for fluid exchange was recognized. This was first proposed to account for the large discrepancies between the lymph flow and the steady state values of Starling forces of various tissues estimated from the mean colloid osmotic pressure of the interstitial fluid demonstrated by Levick (16). This concept, the Michel–Weinbaum model or glycocalyx model, and the associated hockey stick or *J* curve, are discussed in more detail below.

The ***colloid osmotic pressure of plasma*** π_p_ is the major absorptive force. It is readily measurable, though clinicians tend to focus on the plasma albumin concentration as a surrogate (26). There are some misconceptions about albumin and its role in Starling forces. It is correctly taught that plasma albumin accounts for most of the plasma colloid osmotic pressure in most healthy individuals. This is true as albumin accounts for 65% of plasma colloid osmotic pressure but the globulins contribute a not insignificant 35%. Further, there are rare examples of healthy and apparently non-oedematous individuals who are congenitally analbuminaemic. Hypoalbuminaemia is a common finding in critical illness, but is not invariably accompanied by reduced plasma colloid osmotic pressure (27). More importantly, clinical trials show that infusing albumin solutions to maintain a near-normal plasma albumin concentration in patients with severe sepsis does not confer any therapeutic advantage (28).

**Interstitial pressure (*P***_***i***_**)** is the second absorptive Starling pressure. Once believed to be close to or slightly above atmospheric pressure, it is now known to be sub-atmospheric in most tissues of vertebrates where it has been investigated (29, 30). In human subjects, P_i_ is also slightly sub-atmospheric in subcutaneous tissues at heart level (31, 32). Noddeland (31) examined the effects of posture in 10 human subjects and reported a mean value of −1.4 mmHg at approximately heart level and a mean value of −0.4 mmHg at ankle level in standing subjects. The small difference between these mean values were not significant since the range of values was similar (−0.5 to +1.5 mmHg and −0.5 to +4.5 mmHg). There is evidence that P_i_ is more negative in the lungs (33). Acute inflammatory response arising within a tissue can drop the interstitial pressure even more negative, increasing the transendothelial filtration rate. Accumulation of tissue fluid eventually makes the interstitial pressure increasingly positive as lymphatic flow capacity is exceeded.

***Transendothelial resistance to solvent flow*** (*R*_te_) is not of itself a force, it nonetheless describes a characteristic of the microvasculature that explains physiological adjustments and is potentially amenable to therapeutic intervention (10).

Pore theory has been developed into a powerful mathematical model for describing microvascular fluid and solute exchange. The **small pores** are now generally believed to be the spaces between the fibrous molecules of the endothelial glycocalyx overlying the intercellular clefts of continuous endothelia or the fenestrae of fenestrated endothelia (34–36). The numbers of small pores engaged in the filtration process depend upon the number of breaks in the tight junctions which seal the intercellular clefts of continuous endothelia, and the numbers of fenestrae in fenestrated endothelia. The **large pores** are believed to be either rare transendothelial or intercellular channels or possibly transcytotic vesicles (37). An important requirement for the maintenance of the permeability barrier is an uninterrupted supply of sphingosine-1-phosphate which is synthesized in healthy erythrocytes and bound to plasma albumin for transport to the endothelium (38). This important role of albumin in maintaining the capillary barrier requires quite a small concentration of endogenous albumin, and does not justify the administration of pharmaceutical albumin solutions to treat “leaky capillaries.” The older notion that larger molecules should more successfully block large pores is erroneous.

## The Hockey Stick or *J* Curve Relating Steady State Values of Filtration to Microvascular Pressure

Starling believed that microvessels were impermeable to plasma proteins and that the concentrations of proteins in the interstitial fluids were very low. On this basis, he proposed that fluid movements between the plasma and the tissues should be self-limiting (4). For example, raised hydrostatic pressure in capillaries which drives fluid filtration from plasma to tissues concentrates the proteins of the plasma raising its colloid osmotic pressure which opposes filtration until it brings filtration to a halt. He saw this as a rapid means of regulating plasma volume.

Between the 1920s and late 1940s, evidence accumulated to show that proteins passed though capillary walls and were slowly circulating through nearly all the tissues of the body, being returned to the circulation by lymphatics. Apart from the liver, spleen and bone marrow, the permeability of microvessels in most other tissues to plasma proteins is low. It was appreciated that the protein permeability of microvessels meant that the effective colloid osmotic pressure opposing fluid filtration from plasma to tissue was reduced by a factor, the membrane reflection coefficient, σ. For capillaries and venules in most tissues, σ to macromolecules was 0.9 or more, so the effect was relatively small.

If microvascular walls are finitely permeable to proteins, the concentration difference across them, responsible for the colloid osmotic pressure difference, which Starling believed balanced the hydrostatic pressure difference, could not be maintained for more than a fraction of a second. There could be no “Starling equilibrium” because, in the absence of filtration, the colloid osmotic pressure difference would be dissipated by diffusion of protein from plasma to interstitial fluid. It was recognized that the protein concentration differences could be maintained by a low level of filtration since water and small solutes were carried into the interstitial fluid very much faster than large protein molecules. The resulting protein concentration in the ultrafiltrate would be considerably less than that in the plasma. The higher the filtration rate, the lower the concentration of protein in the ultrafiltrate. Consequently, the colloid osmotic pressure difference between plasma and interstitial fluid could be maintained constant by the greater trans-capillary flow rates of the fluid than of protein. While there would be no equilibrium, there could be a steady state difference.

This concept was developed quantitatively in a review of fluid exchange through microvascular walls (39). The steady state values of the colloid osmotic pressure difference depend on the hydrostatic pressures difference driving filtration, the permeability coefficients of the vessel walls and the plasma concentration of proteins. When evaluated for fluid exchange in tissues where permeability coefficients of the microvessels were known, the relation between hydrostatic pressure difference and filtration rate appeared as the “hockey stick” or “*J*” curve. The sharp change (inflection) from low slope to steep slope in most tissues of the systemic circulation occurs just below the colloid osmotic pressure of the plasma. Michel showed that some observations, previously believed to be inconsistent with the Starling Principle, could be accounted for by this steady state curve. The steady state relationship between hydrostatic pressure and filtration rate was later confirmed in experiments on single perfused microvessels where nearly all the variables could be estimated or controlled, for example (40).

**Figure 1:**
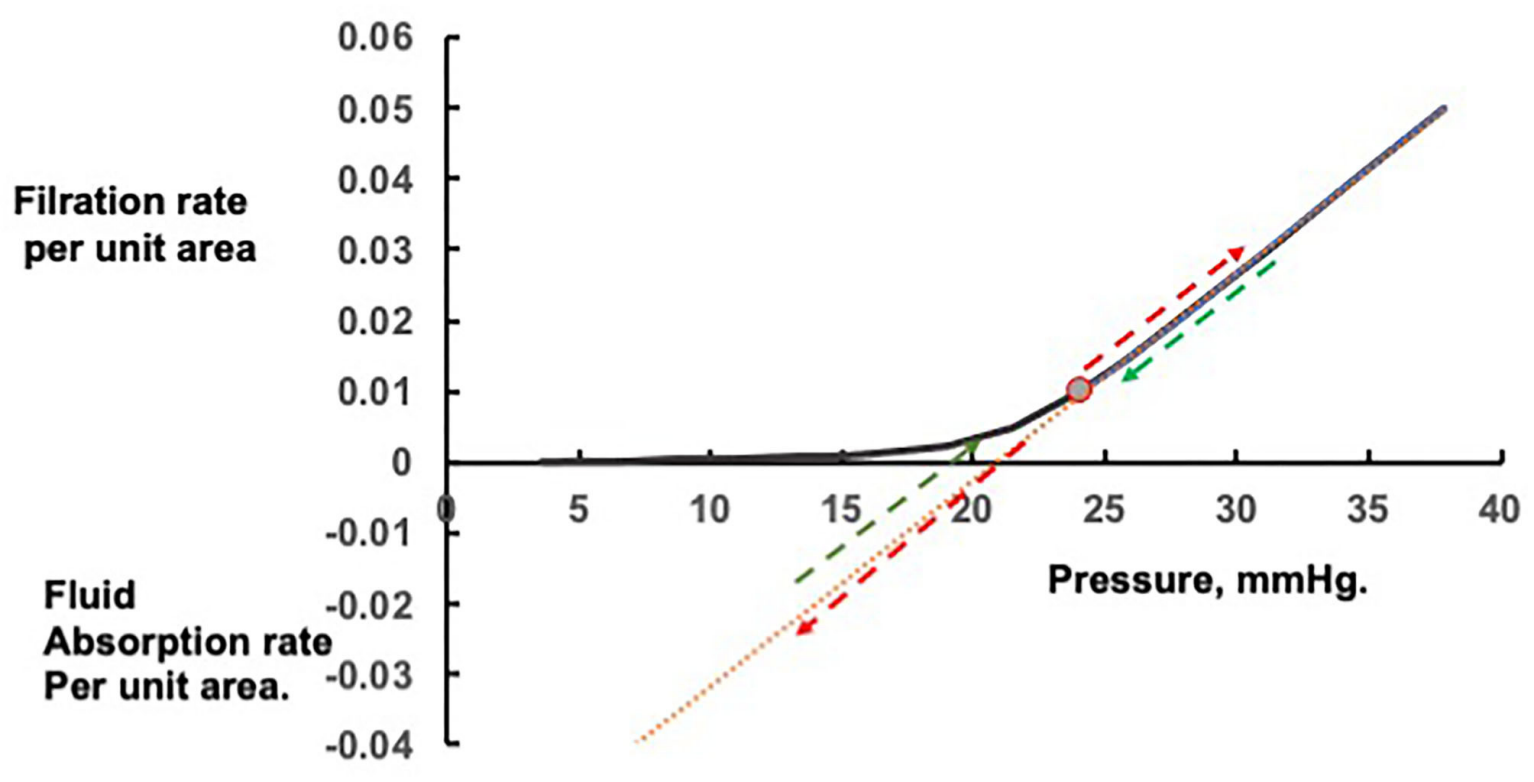
Steady state and transient changes in fluid exchange with changes in microvascular pressure. Rapid changes in fluid filtration and absorption with step changes in pressure from the steady state are linear and indicated by the red arrows; rapid return to the original steady state pressure by the green arrows.

**Figure 2:**
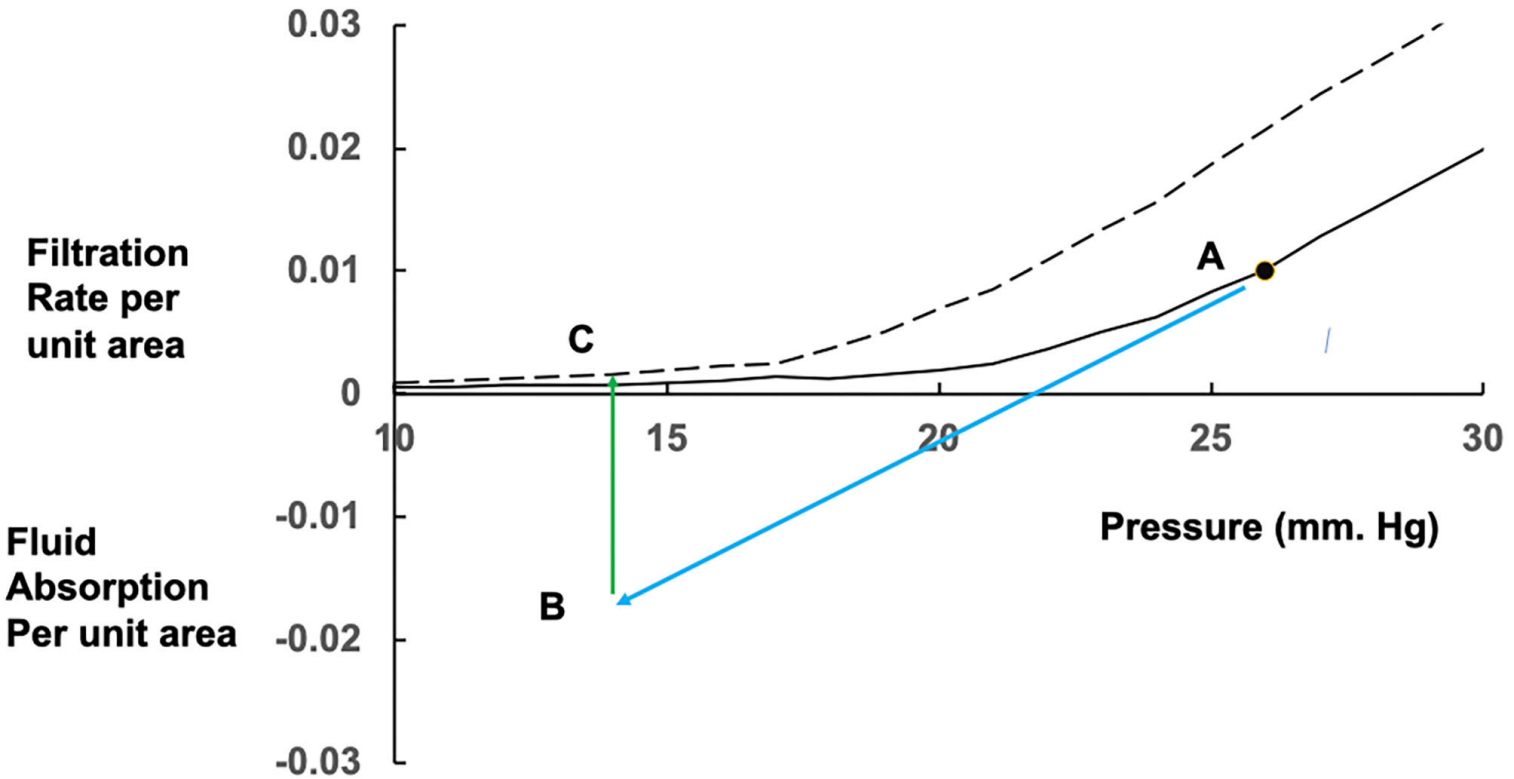
Effects of prolonged fluid absorption on steady state relations between fluid exchange and microvascular pressure. From a position shown as point A on the initial steady state (solid) curve, a fall in mean microvascular pressure leads to a reversal of fluid exchange to fluid absorption (blue arrow) occurring at point B. If the fall in pressure is prolonged, fluid uptake dilutes the plasma protein concentration. Along with changes in the interstitial fluid, the colloid osmotic pressure difference falls, absorption rate falls and eventually reverses to a low level of filtration at point C on the new steady state curve (dashed), which has shifted to the left with the fall in plasma colloid osmotic pressure.

The curve in Figure 1 shows the filtration rates, which will maintain constant values of the colloid osmotic pressure difference across the walls of exchange vessels in a tissue at different microvascular hydrostatic pressures. If the microvascular hydrostatic pressures increase or decrease, transient filtration or fluid uptake will change the effective osmotic pressure differences across the microvascular walls to bring the filtration or reabsorption rates to the level consistent with the new hydrostatic pressure. Often a rise in microvascular hydrostatic pressure in one tissue are offset by simultaneous falls in pressure in another so that protein concentration in the circulating plasma is changed only slightly. If the change in pressure is large and persistent, however, the protein concentration of the plasma circulating through all tissues is changed. In this case, the curve and most conspicuously its inflection point is shifted along the pressure axis. Raised filtration rates in tissues below the heart during lengthy periods of standing concentrate the plasma proteins, raising plasma colloid osmotic pressure and shifting the curve with its inflection region to the right. Prolonged dilution of the plasma, as in auto-transfusion following hemorrhage, or infusion of crystalloid solutions, lower the plasma colloid osmotic pressure and the curve shifts to the left (see Figure 2).

### The *J* Curve as a Clinical Concept

Woodcock proposed a simple paradigm for prescribers of fluid therapy inspired by the physiology described above. He called the inflected relationship between the net microvascular pressure and the whole-body transendothelial filtration rate the *J* curve. In this paradigm we expect the infusion of an intravenous fluid to increase plasma volume, and venous and capillary pressures, resulting in increased transendothelial solvent filtration rate. Of course, in the living animal, there are also endocrine responses to protect homeostasis, that include natriuretic peptide secretion and the renin-angiotensin-aldosterone pathway. If we infuse a solution with no colloid osmotic pressure there is protein dilution and the colloid osmotic pressure of plasma falls, further favoring filtration. If we infuse a hyperoncotic solution of albumin (e.g., 20–25%) the colloid osmotic pressure of plasma (an absorptive force) increases, opposing filtration and shifting the curve to the right.

The *J* curve offers an explanation for the reported phenomenon of context sensitivity. In the face of falling capillary pressure, the transendothelial solvent filtration rate approaches zero, and may reverse to fluid absorption. The astute clinician notices that the colloid osmotic pressure of any resuscitation fluid he administers to a hypovolaemic patient (pressure <18 mmHg in Figure 2, below the *J* point) is of little importance while fluid is no longer leaving the circulation. We now have our explanation for the observed fact that the effective volume of resuscitation with a crystalloid solution is only a little greater than the effective volume of resuscitation with an iso-osmotic colloid solution.

## The Extravascular Circulation of Extracellular Fluid

Spreading the message about this new physiology takes time. In 2010 Levick and Michel made the following plea (41).

*Although doggedly persistent in textbooks and teaching, the traditional view of filtration–reabsorption balance has little justification in the microcirculation of most tissues. Tissue fluid balance thus depends critically on lymphatic function in most tissues. In making these forceful statements, we are mindful of William Harvey's remark in his classic, De Motu Cordis (1628): ‘I tremble lest I have mankind for my enemies, so much has wont and custom become second nature. Doctrine once sown strikes deep its root, and respect for antiquity influences all men’*.

This emotive *cri de couer* led Woodcock and Woodcock to consider consequences and implications for the physiologically rational prescription of intravenous fluids (42), bringing the glycocalyx model into the core knowledge base for clinicians (43). Nonetheless, there remain modern day enemies of this challenge to “wont and custom” (44). There had been previous clues that the Starling principle as taught in the twentieth century could not explain fluid physiology as experienced by clinicians. Cope and Litwin had reported in 1962 that, contrary to Starling principle expectations, plasma volume refill after acute hemorrhage in a canine experiment was attributable to a rise in thoracic duct lymph flow. Lymph flow returned about twice the amount of protein that had been lost by hemorrhage. They called this phenomenon “the essentiality of the lymphatic system to the recovery from shock” (45).

In human volunteer studies published in 1966 F.D. Moore (46, 47) observed that normal transcapillary refill after hemorrhage in man does not involve any significant period of hypoalbuminaemia, which would occur if refilling was solely due to capillary reabsorption of solvent and small solutes. After a 12% reduction in blood volume, the plasma refill rate in these adult male volunteers was about 1 ml per minute. Moore was concerned that crystalloid resuscitation might cause a washout of plasma proteins to the tissues, but in a volume kinetic experiment on the same subjects he found that, while two thirds of infused isotonic salt solution was leaving the circulation, approximately 15–17 g protein (mostly albumin) was entering the plasma. The saline infusion, rather than producing a washout of plasma protein to the interstitium, appeared to restore interstitial fluid volume sufficiently to support protein flow to the plasma. He also presciently warned that “much larger saline infusions, by producing a more drastic protein dilution, might mask this effect completely, leading to the erroneous interpretation of washout.” From the volume kinetics of this crystalloid infusion Moore even determined that the net vector of fluid exchange from plasma to interstitial fluid, and from interstitial fluid to plasma, is about 5 ml per minute. Though he did not realize it, Moore was measuring the extravascular circulatory rate of extracellular fluid. We now know that, in healthy steady state conditions, around 300 ml per hour of solvent and electrolytes leaves the plasma, mostly by transendothelial solvent filtration (48–50), around 8 L per day being absorbed into the lymphatic system (as predicted by William Hunter) and then carried by afferent lymphatic vessels to lymph nodes, where approximately half is absorbed to the blood stream through the diaphragm-fenestrated high endothelia of the lymph node capillaries and venules. The remainder is propelled as efferent lymph to the thoracic duct, and thereby returned to the blood stream in the great veins. Similar studies on species encountered in veterinary practice would be needed to estimate the magnitude of extracellular fluid circulation in them.

The preceding considerations apply to most tissues, most of the time. There are tissues in which there is sustained absorption of fluid from tissue into the blood stream. This is possible because in these tissues, proteins of the interstitial fluid are continually diluted by sources other than filtration from local microvessels. Examples are epithelial secretions into the interstitial fluid of protein free fluid, as in post-glomerular vessels of the kidneys, or the flow of lymph through the interstitium of the lymph nodes. The intestinal microcirculation is able to sustain absorption of ingested fluid when it is available, and to sustain filtration during fasting. Capillaries in such tissues often have circular windows of fused luminal and abluminal endothelial cell membrane; the diaphragm fenestrated capillaries.

**Figure 3:**
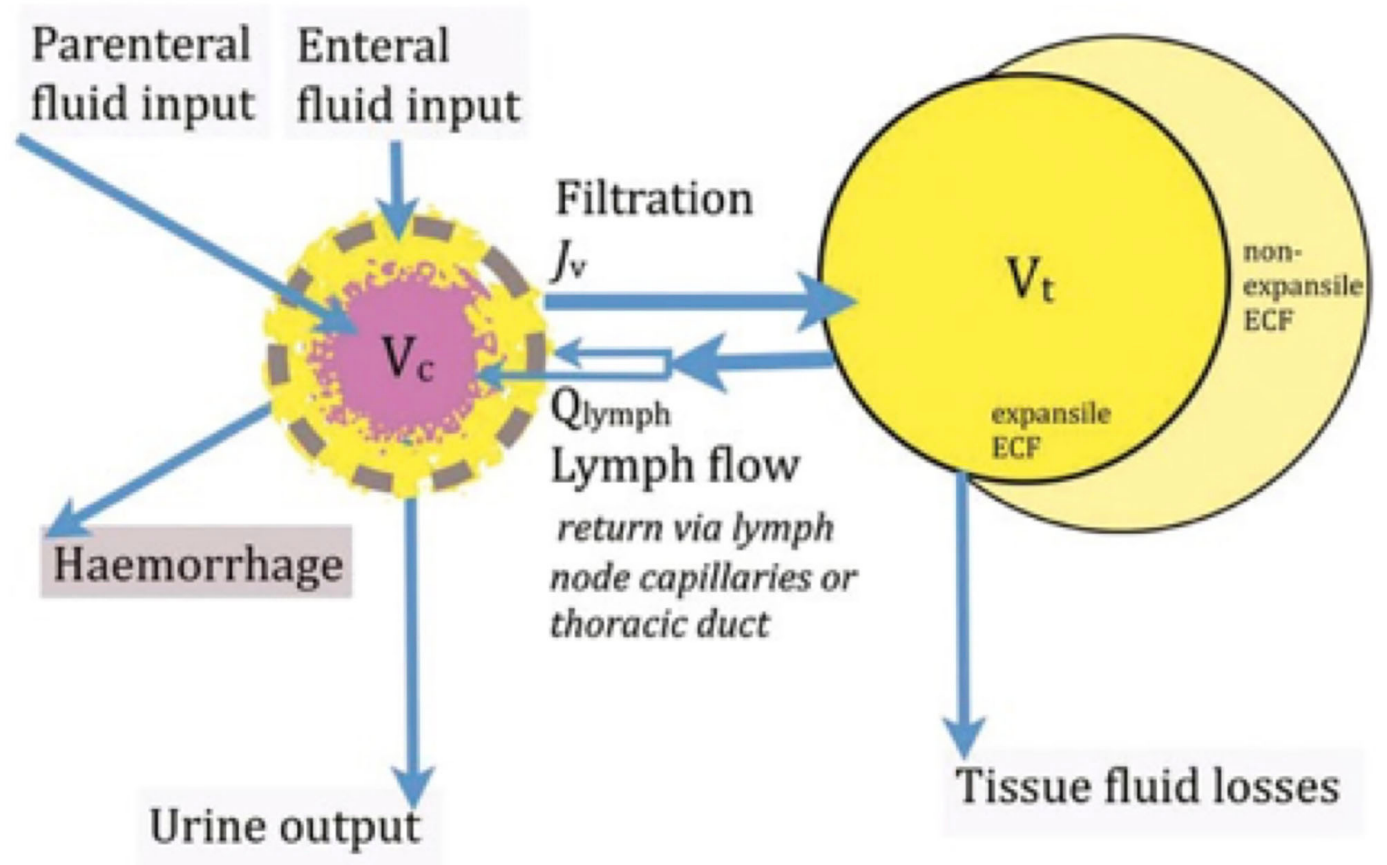
The central volume of distribution Vc of an isosmotic colloid approximates to the free flowing plasma, while the Vc of an isotonic intravenous crystalloid infusion includes the whole of the intravascular space. The tissue volume of distribution Vt of a crystalloid infusion is limited to the expansile tissues, and is much less than the total extracellular fluid (ECF) volume.

Woodcock commends to clinicians a kinetic perspective on the distribution of extracellular fluid between the intravascular and extravascular compartments, revealing ways in which plasma volume can be manipulated therapeutically (10). In Figure 3, the free-flowing plasma is shown in pink, while the intravascular gel phase (the endothelial surface layer), the volume of circulating blood cells and the extravascular gel-phase interstitial fluid is shown in yellow. The central volume of distribution V_c_ of an isoncotic intravenous colloid infusion is largely restricted to the free flowing plasma. The central volume of distribution V_c_ of an isotonic intravenous crystalloid infusion includes the whole of the intravascular space. The tissue volume of distribution Vt of a crystalloid infusion is limited to the expansile tissues, and is much less than the total extracellular fluid (ECF) volume.

## Plasma Volume

As illustrated in Figure 3, plasma volume accumulates by absorption of ingested fluids, the absorption of solvent and small solutes from lymph by lymph node microvessels, and the delivery of efferent lymph to the central veins (51, 52). Our patients face the additional challenge of intravenous fluid infusions. Plasma volume is reduced by urine output and by transendothelial solvent filtration. Surgery, trauma and burns add pathological blood and tissue fluid losses to our considerations. An important contributor to healthy plasma volume homeostasis is the tonic regulation of capillary permeability to albumin. Arteriolar constriction with an infusion of norepinephrine supports plasma volume by reducing capillary pressure so diminishing fluid filtration. Notice that norepinephrine also increases the stressed blood volume, or the effective arterial blood volume if you prefer that concept. In a patient who was not hypovolaemic, norepinephrine infusion can increase urine output. Norepinephrine infusions are increasingly being used in critical care practice to counteract the reduced arterial diastolic pressure and increased capillary pressure associated with deep general anesthesia, epidural anesthesia and systemic inflammatory response. It is obviously vital to avoid higher doses which can lead to tissue ischaemia.

In 1832 Scottish physician Thomas Aitchison Latta pioneered the use of intravenous saline solution for patients dehydrated by cholera. Today we understand that these patients became ill when plasma volume was so reduced that the available venous excess was too small to permit the diastolic right ventricle to fill to normal stroke volume (53, 54). By infusing an isotonic salt solution Latta was resuscitating the intravascular volume, restoring the venous excess volume and increasing the capillary and venular hydrostatic pressures. With reference to the *J* curve, notice that at very low capillary pressure transendothelial solvent filtration and glomerular filtration rates are minimal so that net accumulation of volume is maximized, increasing capillary and venular pressures. Using a colloid resuscitation fluid would be only slightly more efficient than the crystalloid, because preserving plasma colloid osmotic pressure during hypovolaemia has only minimal effect on the transendothelial filtration rate. Because of their lower density and viscosity, crystalloid solutions can be infused more rapidly and will more quickly restore the trans-endothelial and glomerular filtration rates, with greater urine output. The oft-stated logic that “colloids stay in the circulation longer” is misleading; what we need to focus on is the transendothelial and glomerular filtration rate consequences of infusing any intravenous fluid.

There are clinicians whose practice is to infuse fluids to normovolaemic patients with the intention of creating a hypervolaemic hyperdynamic circulatory state, to the point at which further volume expansion is no longer associated with increased stroke volume. For no obvious reason this is often called volume optimisation. Oxygen delivery is often not increased because of dilutional anemia and reduced oxygen content. Referring to the *J* curve, the capillary pressure is rising from normal toward capillary hypertension with increased transendothelial solvent filtration. If this volume expansion is induced with crystalloids, colloid osmotic pressure of plasma is reduced as the capillary hydrostatic pressure rises. Compensation by filtration and return to normovolaemia is therefore rapid. Colloid solutions preserve or even increase the colloid osmotic pressure of plasma, slowing the compensation rate and prolonging the hypervolaemic state. There is no clinical evidence that this practice is of greater benefit to patients than other perioperative fluid management strategies (54).

In modern perioperative care the available evidence suggests the optimal goal for fluid balance is a modest positive balance which guards against intravascular hypovolaemia, with minimal harm from oedema or heart failure. There is no evidence that the use of synthetic colloids or albumin is necessary to achieve optimal patient outcome (54).

## A New Physiologically-Based Paradigm for Rational Fluid Therapy Prescription

**Figure 4:**
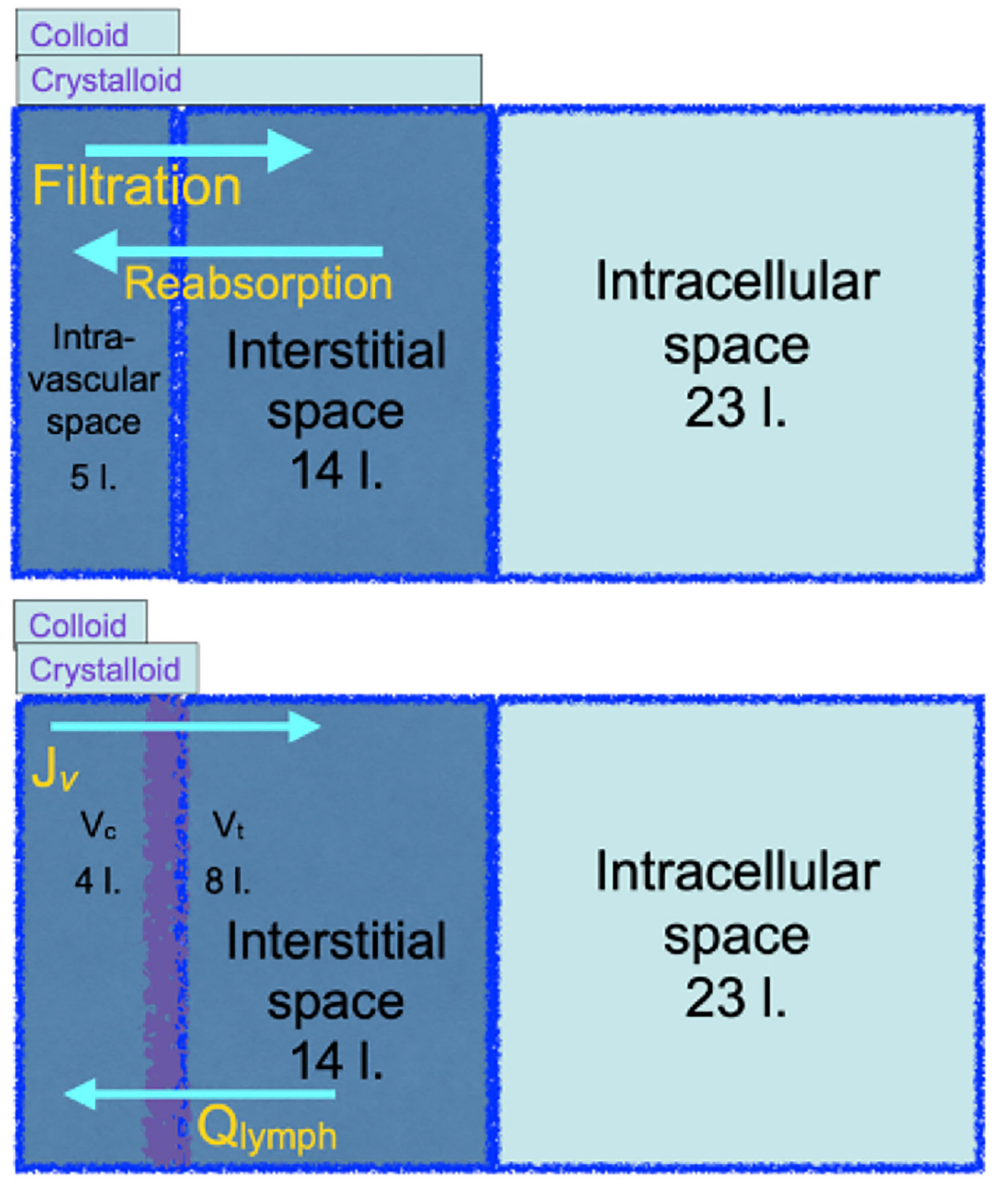
The top cartoon is a familiar illustration of the distribution of body water in original Starling physiology theory that implies that crystalloid solutions will be inefficient for resuscitation from a reduced plasma volume. The bottom cartoon, grounded in revised Starling physiology, explains the observed relative efficiencies of either colloid or crystalloid resuscitation.

In 1985 Twigley and Hillman announced ‘the end of the crystalloid era’ (55). Using a simplified diagram of plasma, interstitial and intracellular fluid compartments, and their anatomic volumes, they argued that colloids could be used to selectively maintain the plasma volume. Plasma volume being about 20% of the extracellular fluid (ECF), it was presumed that the volume equivalence for resuscitation from intravascular hypovolaemia would be of the order of 100 ml isotonic salt solution to 25 ml colloid. Moreover, it was presumed from Starling's principle that transfusion of hyperoncotic colloid solutions would sustain absorption of fluid from the interstitial fluid to the intravascular volume. This simple concept of colloid for plasma volume and isotonic salt solution for extracellular fluid replacement has been continued and developed. For adult human body water, see the top diagram, Figure 4.

Large randomized clinical trials reported in the twenty first century to date unanimously reject the concept of crystalloid inefficiency as a resuscitator from reduced plasma volume. The bottom diagram, Figure 4, updates the familiar body water diagram, with the expected central volumes of distribution of a colloid or a crystalloid infusion. The SAFE study of 2004 (56) looked at critically ill patients over the first 4 days of fluid resuscitation. 100 ml isotonic salt solution was as effective as 62–76 ml human albumin solution in the SAFE study, or 63–69 ml hyperoncotic plasma substitute in VISEP (57). The FIRST study enrolled blunt trauma patients during the first day of resuscitation, in whom 100 ml isotonic salt solution was as effective as 97 ml isosmotic plasma substitute, while in gunshot or stabbing victims, 100 ml was as effective as 67 ml (58). After Woodcock's paradigm was published in 2012, three further trials were published. All reported the volume equivalence for resuscitation of hydroxyethyl starch to 100 ml isotonic salt solution. The CHRYSTMAS study averaged 80 ml (59), the CHEST study 85 ml (60), and the 6S trial reported the median cumulative volume of fluid received was 3,000 ml in both groups (61). The FLASH randomized controlled trial gave resuscitative equivalents of 100 ml saline to be 83 ml hydroxyethyl starch (62). It is often noted that the colloid treated patients receive more blood transfusions, which the Woodcock paradigm attributes to greater haematocrit reduction by colloids. In the ALBIOS study, administration of human albumin solution to keep the plasma albumin concentration above 30 g/ l did not reduce the intravenous fluid volume administered and had no effect on patient outcome (28).

The new formulation of the century old Starling principle inspired a new clinical paradigm for fluid therapy (Table 1) that draws on the extended Starling principle and an appreciation of all the Starling forces, and understands the many limitations of biophysical colloid osmotic pressure therapy. It reminds us of the importance of protecting and even enhancing lymphatic pump efficiency to boost the plasma volume. It suggests ways in which alpha-adrenergic agonists, and perhaps other vasoactive agents, can be prescribed in doses that optimize capillary and venular pressures for circulatory blood flow and transendothelial solvent filtration (63). New avenues of research are needed (64).

**Table 1:** A comparison of clinical expectations based upon the Original or Extended Starling principle paradigms.

**Original starling principle paradigm**	**Extended starling principle or glycocalyx model paradigm**
Intravascular volume consists of plasma and cellular elements.	Intravascular volume consists of glycocalyx volume, plasma volume, and cellular elements.
Capillaries separate plasma with high protein concentration from interstitial fluid (ISF) with low protein concentration.	Sinusoidal tissues (marrow, spleen, and liver) have *discontinuous capillaries* and their interstitial fluid (ISF) is essentially part of the plasma volume. *Open fenestrated capillaries* produce the renal glomerular filtrate. *Diaphragm fenestrated capillaries* in tissues with an independent supply of fluid to the interstitium can sustain absorption of ISF to plasma. *Continuous capillaries* exhibit ‘no absorption’. The endothelial glycocalyx is semi-permeable to proteins and their concentration in the microdomain below the glycocalyx varies with transendothelial solvent filtration (*J*v).
The important Starling forces are the transendothelial pressure difference and the plasma–interstitial colloid osmotic pressure difference operating across a porous endothelial barrier.	The important Starling forces are the transendothelial pressure difference and the plasma – subglycocalyx colloid osmotic pressure difference operating across the continuous glycocalyx.
Fluid is filtered from the arterial end of capillaries and absorbed from the venular end, while a small proportion returns to the circulation as lymph.	Transendothelial solvent filtration (*J*v) is much less than predicted by Starling's principle, and the major route for return to the circulation is as lymph.
Raising plasma colloid osmotic pressure enhances absorption and shifts fluid from ISF to plasma.	Raising plasma colloid osmotic pressure reduces *J*v but does not cause sustained absorption of ISF. Auto transfusion after abrupt disequilibrium is a transient and limited phenomenon.
At subnormal capillary pressure, net absorption increases plasma volume.	At subnormal capillary pressure, *J*v approaches zero. Auto transfusion after abrupt disequilibrium is a transient phenomenon, and limited to about 500 ml.
At supranormal capillary pressure, net filtration increases ISF volume.	At supranormal capillary pressure, when the colloid osmotic pressure difference is maximal, *J*v is proportional to the transendothelial pressure difference.
Infused colloid solution is distributed through the plasma volume, and infused isotonic salt solution through the extracellular volume.	Infused colloid solution is initially distributed through the plasma volume, and infused isotonic salt solution through the intravascular volume. At supranormal capillary pressure, infusion of colloid solution preserves plasma colloid osmotic pressure, raises capillary pressure, and increases *J*v. At supranormal capillary pressure, infusion of isotonic salt solution also raises capillary pressure, but it lowers colloid osmotic pressure and so increases *J*v more than the same colloid solution volume. At subnormal capillary pressure, infusion of colloid solution increases plasma volume and infusion of isotonic salt solution increases intravascular volume, but *J*v remains close to zero in both cases.

## Author Contributions

TW was invited to contribute this narrative review to be one in a themed series and is responsible for interpretation of the consequences and implications of Starling physiology for fluid therapy, created Figures 3 and 4. CM developed the explanation of Starling physiology as presented here and created Figures 1 and 2. All authors contributed to the article and approved the submitted version.

## Conflict of Interest

The authors declare that the research was conducted in the absence of any commercial or financial relationships that could be construed as a potential conflict of interest.
